# Timely Inhibition of Notch Signaling by DAPT Promotes Cardiac Differentiation of Murine Pluripotent Stem Cells

**DOI:** 10.1371/journal.pone.0109588

**Published:** 2014-10-14

**Authors:** Yinan Liu, Peng Li, Kaiyu Liu, Qihua He, Shuo Han, Xiaofeng Sun, Tao Li, Li Shen

**Affiliations:** 1 Stem Cell Research Center, Department of Cell Biology, School of Basic Medical Sciences, Peking University, Haidian District, Beijing, China; 2 Department of Biochemistry and Molecular Biology, School of Basic Medical Sciences, Peking University, Haidian District, Beijing, China; 3 Center of Medical and Health Analysis, School of Basic Medical Sciences, Peking University, Haidian District, Beijing, China; 4 Department of histology and embryology, Institute of Chinese Medicine, Hunan University of Chinese Medicine, Science Garden District of Hanpu, Changsha, Hunan, China; 5 Department of Biology, College of Chemistry and Life Sciences, Zhejiang Normal University, Jinhua, Zhejiang, China; University of Kansas Medical Center, United States of America

## Abstract

The Notch signaling pathway plays versatile roles during heart development. However, there is contradictory evidence that Notch pathway either facilitates or impairs cardiomyogenesis *in vitro*. In this study, we developed iPSCs by reprogramming of murine fibroblasts with GFP expression governed by *Oct4* promoter, and identified an effective strategy to enhance cardiac differentiation through timely modulation of Notch signaling. The Notch inhibitor DAPT (N-[N-(3,5-difluorophenacetyl)-l-alanyl]-S-phenylglycine t-butyl ester) alone drove the iPSCs to a neuronal fate. After mesoderm induction of embryoid bodies initiated by ascorbic acid (AA), the subsequent treatment of DAPT accelerated the generation of spontaneously beating cardiomyocytes. The timed synergy of AA and DAPT yielded an optimal efficiency of cardiac differentiation. Mechanistic studies showed that Notch pathway plays a biphasic role in cardiomyogenesis. It favors the early–stage cardiac differentiation, but exerts negative effects on the late-stage differentiation. Therefore, DAPT administration at the late stage enforced the inhibition of endogenous Notch activity, thereby enhancing cardiomyogenesis. In parallel, DAPT dramatically augmented the expression of *Wnt3a*, *Wnt11*, *BMP2*, and *BMP4*. In conclusion, our results highlight a practicable approach to generate cardiomyocytes from iPSCs based on the stage-specific biphasic roles of Notch signaling in cardiomyogenesis.

## Introduction

Induced pluripotent stem cells (iPSCs) are novel pluripotent stem cells generated from adult tissues by reprogramming with the transduction of a defined set of transcription factors [1], [2]. iPSCs can be propagated indefinitely, and possess the potential to differentiate toward triploblastic function-cells including cardiomyocytes and vessel cells both *in vitro* and *in vivo*. However, the conventional approach to acquire differentiated cardiomyocytes from iPSCs using the hanging drop method is severely inefficient, which poses a major obstacle to the use of iPSCs for cardiac repair. It is indispensable to optimize methods that induce the differentiation of iPSCs into mature and functional cardiomyocytes. Notably, the culture of stem cells as embryoid bodies simultaneously with the supplementation of various growth factors (such as BMP2, BMP4, Activin A, bFGF, FGF10, and Wnt3a), has also been found to improve the induction efficiency of spontaneously beating cardiomyocytes and appears to be a common approach [3]–[8]. These protocols are all limited for large-scale applications due to low throughput and expensive outlay of protein inducing reagents.

Recently, many efforts have focused on screening small molecules to improve the efficiency of iPSC differentiation into cardiomyocytes. Using a high-throughput screening system, ascorbic acid (AA) was found to robustly and reproducibly simulate the generation of cardiomyocytes from pluripotent stem cells [9]. The induction methodology utilizing AA has been successfully applied in the cardiac differentiation of ESCs and iPSCs [10]. Mechanistic study showed that AA stimulated the proliferation of cardiac progenitor cells originated from stem cells via the MEK-ERK1/2 pathway by promoting collagen synthesis, leading to an expanded pool of committed progenitors destined to acquire a cardiac fate [10], [11]. Moreover, a combination of AA and Salvianolic acid B yielded a synergy promoting cardiac differentiation, which points to a possible approach using optimized small molecule cocktails to generate cardiomyocytes from various stem cells [12].

Notch is an evolutionary conserved pathway with important functions in various events during development, including cell communication, maintenance of tissue boundaries, cell fate acquisition, and differentiation. Notch is a single-pass transmembrane receptor which is activated when its extracellular domain binds with ligands in a neighboring cell. Four Notch receptors (Notch1–4) and five Notch ligands (Jagged-1, -2, and Delta-1, -3, -4) have been identified in mammals. Upon ligand binding to Notch receptor, the receptor undergoes two sequential proteolytic cleavages. The latter cleavage at the transmembrane domain is accomplished by the γ-secretase enzyme complex and yields the Notch intracellular domain (NICD), which translocates to the nucleus and activates transcription via interacting with RBP-J, which is responsible for targeting NICD to specific promoters. The best-characterized transcriptional targets of Notch include the *Hes* and *Hey* family genes. Both the *Hes* and *Hey* families function as transcriptional repressors with basic helix-loop-helix (bHLH) domain, regulating progenitor cell fate in multiple tissues. Meanwhile, there are several putative Notch targets, including *c-Myc*, *Sox9*, *Pax6*, *Runx1*, *Myf5*, *Mash1*, *MyoD*, *Nkx2.5*, and *GATA2*, which are critically involved in stem cell renewal or lineage decision [13]. It implies that Notch can play pleiotropic roles in diverse stages of heart development [14]–[16]. Thus, small molecules targeting Notch signaling might be an approach for generating large numbers of cardiomyocytes from pluripotent stem cells. However, there were also conflicting evidences, stating that Notch activation can both promote and inhibit cardiac differentiation [17]–[21].

In the present study, we developed iPSCs from murine fibroblasts and induced cardiomyogenesis through formation of embryoid bodies (EBs). We evaluated the effect of Notch inhibition by DAPT on cardiac differentiation of murine iPSCs, and discovered that later treatment with DAPT after mesoderm induction triggered by AA markedly accelerated the production of spontaneously beating cardiomyocytes. Nevertheless, persistent inhibition of Notch signaling by DAPT beginning from EB formation specifically favored iPSCs differentiation into neuronal differentiation. Combined with data from Notch1 RNAi and NICD1 overexpression, our results showed that Notch pathway plays a stage-specific biphasic role in cardiomyogenesis. Our study provided an easily accessible approach to efficiently generate cardiomyocytes from iPSCs via timely inhibition of Notch signaling using small molecules.

## Materials and Methods

### Ethics statement

The animal experiments were carried out in strict accordance with the recommendations in the Guide for the Care and Use of Laboratory Animals of the National Institutes of Health. The protocol was approved by the Committee on the Ethics of Animal Experiments of the Peking University (LA201485). All surgery was performed under sodium pentobarbital anesthesia, and all efforts were made to minimize suffering.

### Cell culture

Mouse embryonic fibroblasts (MEFs) with GFP driven by *Oct4*-promoter were kindly provided by Pro. Hongkui Deng (College of Life Sciences, Peking University). MEFs and HEK293T cells were maintained in DMEM high glucose (GIBCO, USA) supplemented with 10% FBS (Hyclone, USA). iPSCs and ESCs were maintained in a standard ESC medium containing 15% FBS, 1% non-essential amino acids, 2 mM glutamine, 0.1 mM β-mercaptoethanol, 100 U/mL penicillin, and 100 mg/mL streptomycin (Hyclone, USA) on mitomycin C (Sigma, USA)-treated MEF feeder layers in the presence of leukemia inhibitory factor (Millipore, USA, 1000 U/mL). Colonies were dissociated using trypsin and passaged at a 1∶3 split ratio every 3–4 days depending on the cell density.

### Generation of induced pluripotent stem cells

The generation and structure of doxycycline (Dox)-controlled Tet-on-inducible lentiviruses expressing Yamanaka factors have been described in previous reports [22]. To produce lentiviral particles used for reprogramming, TetO-FUW lentiviral vectors (Addgene, www.addgene.org) encoding the mouse cDNAs of *Oct4*, *Sox2*, *Klf4*, and *c-Myc* were cotransfected into 293T cells along with lentivirus packaging plasmids ps-PAX-2 and pMD2G using Lipofectamine 2000 (Invitrogen, USA). Viral supernatant was harvested at two time points, 24 h and 48 h after transfection. Typically, 38 mL of supernatant was collected per virus. Viral supernatant was filtered through a 0.45 mm syringe filter (Millipore, USA), and concentrated by ultracentrifugation at 25,000 rpm for 2 h. Titration of virus was measured by FACS assay using EGFP expressed lentivirus according to the formula: GFP^+^ % × target cell numbers/virus transduced volume (TU/mL) as described before [23]. MEF cells of embryos of 14.5 days were obtained from *Oct4*-promoter driven GFP transgenic mice [24]. MEFs at a density of 2×10^4^ cells/plate were infected with lentivirus (2–5 µL of each viral concentrate) two times in the presence of 8 µg/mL polybrene (Sigma, USA), and the medium was replaced 24 h after infection. After 2 days of viral infection, the culture medium was replaced by mESC medium supplemented with 2 mg/mL Dox to induce reprogramming. The newly generated mouse iPSC colonies were picked up after 9–12 days.

### Characterization of iPSCs

The isolation of total RNA and RT-PCR of marker gene expression were performed as described by Takahashia K et al [1]. The primer sequences were shown in the Table S1. For immunofluorescence assay, cells were fixed with 4% paraformaldehyde for 10 min at room temperature. After being washed with PBS, the cells were treated with PBS containing 10% normal bovine serum albumin (Sigma, USA) and 0.1% TritonX-100 for 30 min at room temperature, and then incubated with primary antibodies overnight at 4°C. Primary antibodies included SSEA-1 (Chemicon, USA, 1∶100), Nanog (Abcam, UK, 1∶100), Oct4 (Santa Cruz, USA, 1∶100). Subsequently secondary antibodies of anti-rabbit or anti-mouse IgG conjugated with fluorescein (Santa Cruz, USA, 1∶200) were incubated to visualize the pluriopotent markers by confocal microscopy (Leica TCS SP5, Germany). Nuclei were counterstained with Hoechst33342. Alkaline phosphatase staining using the alkaline phosphatase kit (Millipore, USA) was performed according to the manufacturer’s instructions. To test the *in vivo* pluripotency, teratoma formation was performed. In brief, iPSCs were harvested, suspended in PBS, and injected subcutaneously into the posterior limbs of BALB/c SCID mice (3×10^6^ cells per mouse). Around four weeks after injection, tumors were dissected, fixed in 4% paraformaldehyde, embedded in paraffin, and examined histologically by hematoxylin and eosin staining. The chromosomal G-band analyses were performed in the Cytogenetics Laboratory at Peking University.

### Induction differentiation and immunofluorescence identification

In order to ensure proper formation of embryoid bodies (EBs), hanging drop for generating EBs was used. The iPSCs were detached by trypsinization and resuspended in ESC medium without LIF factors. After cells at a dilution of 25,000 cells/mL were counted, 20 µL cell solution drop containing 500 cells was plated on the cover of 100 mm dishes that were filled with 5 mL PBS to prevent evaporation and incubated for 2 days. On the third day, the EBs were harvested and transferred to bacterial petri dishes for another two days. On day 5, EBs were attached to plates coated with 0.1% gelatin and maintained in DMEM high glucose supplemented with 5% FBS, 200 µM L-Glutamine, 1% non-essential amino acids, 0.1 mM β-mercaptoethanol, 100 U/mL penicillin, and 100 mg/mL streptomycin (designated as induction medium). The medium was refreshed every 2 days. AA (Sigma, USA, 100 µM) or DAPT (Sigma, USA, 10 µM) or AA plus DAPT were added to induce differentiation at the indicated time point.

For immunofluorescence identification of differentiated cardiomyocytes, iPSCs were replated on coverslips and induced until day 12 to 16, and then fixed using the above described method. The primary antibody against sarcomeric α-actinin (Sigma, USA, 1∶200) or troponin T (Abcam, UK, 1∶100) was used. Quantitative evaluation of differentiation efficiency was performed by counting troponin T-positive cells. A minimum of five randomly imaged fields of each coverslip was counted from at least five coverslips. The percentage of troponin T-positive cells out of the total number of cells counted represented the differentiation efficiency. Antibodies against Nestin (Sigma, USA, 1∶100) and Pax6 (Abcam, UK, 1∶100) were used to distinguish iPSC-derived neuronal cells by immunofluorescent staining.

### RT-RNA and real-time PCR

Total RNA was extracted using Trizol reagent (Invitrogen, USA) according to the manufacturer’s instructions. Approximately 2 µg of total RNA from each sample was used for random-primer reverse transcription (Invitrogen, USA). For RT-PCR, Taq DNA polymerase (Promega, USA) and Gene-Amp PCR System 9700 (Applied Biosystems, USA) were utilized. The following thermal profile was used for all PCR experiments: 95°C for 5 min, and then appropriate cycles at 95°C for 30 sec, annealing temperature (Table S1, S2, S3) for 30 sec, and 72°C for 30 sec; and terminated by a final extension at 72°C for 8 min. For real-time RT-PCR, MX3000p sequence detector (Agilent Technologies, USA) and SYBR Green real-time Master Mix (Toyobo, Japan) were used. Relative PCR signals were normalized to the average expression levels of the undifferentiated samples. Primers for RT-PCR and real-time PCR are listed in Table S2 and Table S3.

### Immunoblot analysis

Total proteins were extracted from iPSCs or differentiating EBs at indicated time points, and subjected to SDS-PAGE electrophoresis followed by transfer onto nitrocellulose membranes (Amersham, UK), which were then blocked in 5% nonfat milk and incubated with specific primary antibodies, including anti-sarcomeric a-actinin (Sigma, USA), anti-Nanog (Millipore, USA), anti-Oct4, anti-Sox2, anti-Notch1 (Santa Cruz, USA), anti-β-catenin (Santa Cruz, USA) and anti-β-actin (Chemicon, USA). Immunodetection was performed using IRDye700 and 800-conjugated secondary antibodies (Rockland, USA) according to the manufacturer’s instructions. The membrane was scanned using the Odyssey infrared imaging system (Li-cor, USA) at 700 or 800 channel wave length.

### Plasmids transfection and RNA interference

The iPSCs were transfected with plasmids or synthesized siRNA for gene overexpression or knockdown, respectively. The plasmid carrying Notch1 active region NICD-1 (pCDNA3.1-NICD-1) was kindly provided by Dr. Zhuqing Jia (Department of Biochemistry and Molecular Biology, School of Basic Medical sciences, Peking University). pCDNA3.1 vector was used as a control. Nonsilencer siRNA (sense: UUCUCCGAACGUGUCACGUTT; antisense: ACGUGACACGUUCGGAGAATT) and Notch1 siRNA (RNAi-1 sense: CUCCAACCCAUGUCAGAAUTT, antisense: AUUCUGACAUGGGUUGGAGTT, RNAi-2 sense: GCAUCAGCCACUUGAAUGUTT, antisense: ACAUUCAAGUGGCUGAUGCTT, RNAi-3 sense: GGGCUAUGAAUUUCACCGUTT, antisense: ACGGUGAAAUUCAUAGCCCTT) duplexes were synthesized by Shanghai Genepharma Co., Ltd. Transfection of the plasmids or siRNA was performed using Lipofectamine 2000 or Lipofectamine RNAiMAX according to the manufacturer’s instructions (Invitrogen). After 24 h of transfection, the cells were digested into single cells and then cultured in hanging drops to induce cardiac differentiation with AA. The cells were harvested at the indicated days of differentiation for real-time PCR or immunoblot analysis.

### Statistical Analysis

All quantitative data were presented as mean values plus or minus s.d. The statistical significance of compared measurements was assessed using the Student’s t test analysis of variance and *p* values were as follows: *p<0.05; **p<0.01.

## Results

### Reprogramming of MEF cells into iPSCs

**Figure 1:**
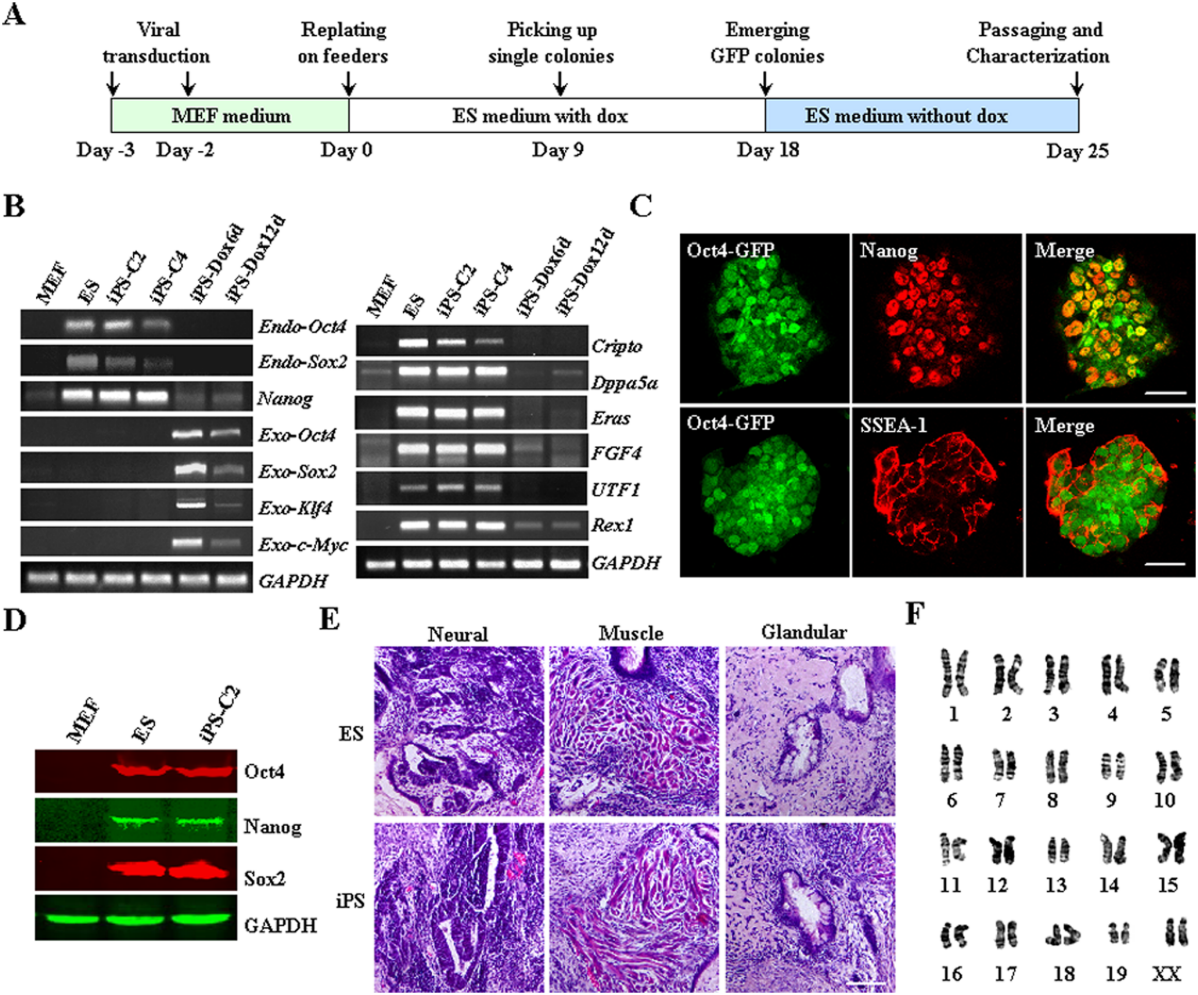
Derivation of iPSCs using an inducible lentiviral system. (**A**) Schematic representation of iPSC generation. (**B**) RT-PCR analysis of exogenous Yamanaka factors, ES pluripotent markers in fully and partially reprogrammed iPSCs (iPS-Dox6d, iPS-Dox12d). Parental MEFs and ESCs were used as negative and positive controls. (**C**) Immunofluorescence analysis of pluripotent markers, Nanog and SSEA-1 (Red). Oct4-GFP^+^ cells (Green) represented fully reprogramming iPSCs. Scale bar 50 µm. (**D**) Western blot analysis confirmed the pluripotent properties of iPSCs expressing Oct4, Nanog and Sox2. Parental MEF and ES cells were used as negative and positive controls. (**E**) Teratoma formation of iPSCs transplanted into immunodeficient mice. After 4 weeks, iPSC-derived tumors were sectioned and stained with hematoxylin and eosin. Shown were neural (ectoderm), muscle (mesoderm) and glandular (endoderm) tissues from left to right. Scale bar 100 µm. (**F**) Karyotyping analysis showing normal karyotyping of iPSCs after passage 10.

MEF cells from transgene mice with GFP driven by *Oct4*-promoter were transduced two rounds with lentiviruses of Yamanaka factors. At day 0, the cells were trypsinized, plated onto mitotically inactivated feeder cells and cultured using ESC medium supplemented with 2 µg/mL Dox (Fig. 1A). After continuous culture for 18 days, approximately 25 colonies with visible GFP fluorescence emerged from 2×10^4^ original cells. These colonies exhibited distinct ESC-like morphology and stained positive for alkaline phosphatase (Fig. S1A). Two of these colonies stably expressing GFP after the removal of Dox were picked and propagated for further experiments, designated as C2 and C4 respectively (Fig. S1B). RT-PCR analysis revealed that the two colonies expressed endogenous *Oct4*, *Sox2* as well as other ESC markers, such as *Nanog*, *Cripto, Dppa5a, Eras*, *FGF4*, *UTF1* and *Rex1,* while the exogenous Yamanaka factors were silenced (Fig. 1B). These cells from colonies picked were positive for Nanog and SSEA-1 immunofluorescence staining (Fig. 1C). Western blot analysis further confirmed the sustained expression of typical pluripotent markers such as Oct4, Sox2, and Nanog in GFP-positive cells, as was the case with ES cells but in contrast to the parental MEF cells (Fig. 1D). Subcutaneous injection of these iPSCs into BALB/c SCID mice led to teratoma formation with three germ layers, fulfilling the definition of pluripotency (Fig. 1E). Meanwhile, karyotype analysis after passage 10 showed that the cells maintained genetic stability (Fig. 1F). Taken together, our results demonstrated that MEF cells were successfully reprogrammed to iPSCs.

### Ascorbic acid induces cardiac differentiation of iPSCs

**Figure 2:**
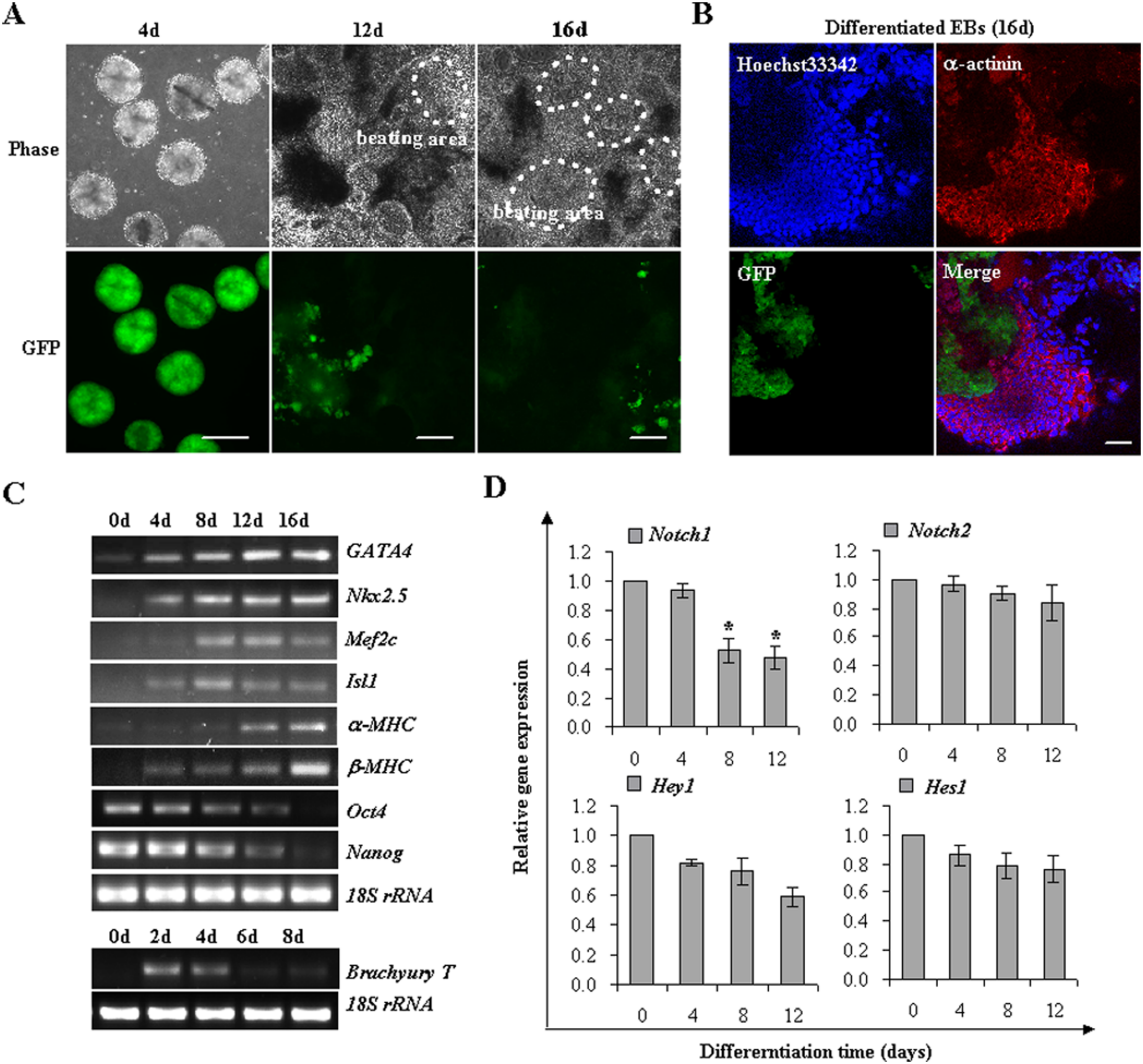
AA induces cardiac differentiation of iPSCs. (**A**) iPSCs were cultured in hanging drops and induced with AA. With the disappearance of Oct4-drived GFP expression, beating cardiomyocytes were observed at day 12 and increased until day 16 of iPSC differentiation, as indicated by closed dashed lines. Scale bar 50 µm. (**B**) Immunofluorescence analysis of iPSC-derived cardiomyocytes at day 16 with a monoclonal antibody against sarcomeric α-actinin (Red). Undifferentiated iPSCs were identified by GFP expression droven by *Oct4-*promoter (Green). Scale bar 50 µm. (**C**) Cardiac-specific gene expression profiles were analyzed by RT-PCR during iPSC differentiation at indicated time points. *18S rRNA* expression was used as an internal control. (**D**) Relative expression levels of several Notch pathway members were analyzed by real-time RT-PCR. Data are shown as relative gene expression with means ± s.d (n = 3) (*p<0.05 vs control group at day 0).

To characterize the differentiation potential into cardiomyocytes, the iPSCs were induced with AA, an ideal cardiomyocyte inducer independent of its antioxidative property. iPSCs were cultured in hanging drops with AA administration and aggregated to form embryoid bodies (EBs). At day 5, EBs were plated for adherent cultivation. After AA treatment for 12 days, spontaneous and rhythmic contractions were initially observed. The spontaneously beating areas were vigorously expanded at day 16 (Fig. 2A). The cardiac differentiation of iPSCs was further confirmed by positive staining of sarcomeric α-actinin (Fig. 2B). RT-PCR demonstrated the appearance of typical cardiac markers, such as *GATA4*, *Nkx2.5*, *Mef2c*, *Isl1*, *α-MHC*, and *β-MHC* in a developmentally sequential manner after AA induction. *Oct4* and *Nanog* mRNA, highly expressed in undifferentiated iPSCs, gradually diminished during the differentiation process. The mesodermal marker, *Brachyury T*, was transiently expressed from day 2 to 4 (Fig. 2C). Thus, the iPSCs in EB system successfully differentiated into functional cadiomyocytes, offering a reproducible platform for the optimization of signaling pathways for efficient cardiac induction.

Notch signaling represents an important regulator of stem cell renewal and differentiation. Notably, some key components of Notch pathway, including *Notch1*, and *Hey1*, showed a decreased expression pattern during the differentiation process, while no significant changes were observed in *Notch2* and *Hes1* (Fig. 2D). Manipulating the activity of Notch signaling may contribute to improve cardiac differentiation efficiency.

### DAPT alone facilitates neuronal differentiation of iPSCs

**Figure 3:**
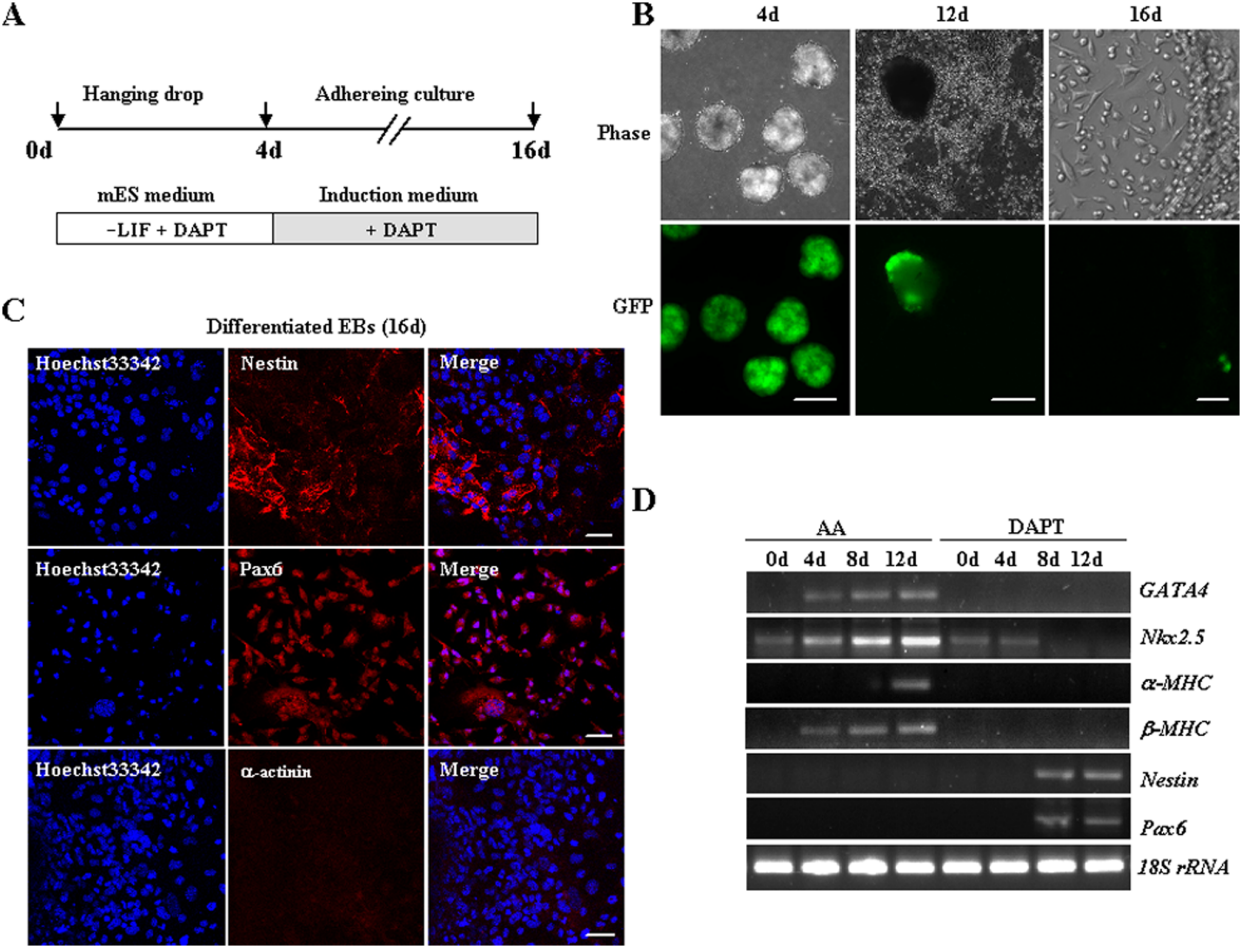
DAPT alone instructs neuronal differentiation of iPSCs. (**A**) Schematic diagram of differentiation protocol with DAPT. (**B**) Differentiated iPSCs induced by DAPT alone exhibited neural phenotypes with a more spindle-shaped morphology from day 12 to day 16. Scale bar 50 µm. (**C**) Immunofluorescence analysis of DAPT-treated iPSCs at day16 with neural (Nestin and Pax6) or cardiac markers (α-actinin). Nuclei were counterstained with Hoechst33342 (blue). Scale bars 50 µm. (**D**) RT-PCR analysis for cardiac markers (*GATA4*, *Nkx2.5*, *α-MHC*, *β-MHC*) as well as neural markers (*Nestin*, *Pax6*) during DAPT induction at indicated time points. *18S rRNA* expression was used as an internal control. Representative results were from three independent experiments.

Previous studies suggested that Notch blockade could accelerate the mesodermal and cardiac decision of ESCs [18]. Subsequently, we estimated the effect of DAPT on cardiac differentiation of the iPSCs. DAPT, an inhibitor of γ-secretase, consequently prevents the proteolysis of Notch receptors and suppresses the Notch activity. However, durative treatment of EBs derived from the iPSCs with DAPT largely enhances the cell fate transition towards neuronal commitment rather than cardiomyocytes. In our current research, after exposure to DAPT for 12 to 16 days, differentiated cells adopted a typical neuronal morphology such as discernible outgrowth of protrusions (Fig. 3A, 3B). A substantial proportion of EBs was positively stained with Nestin and Pax6, widely employed markers of multipotent neural progenitor cells at day 16 (Fig. 3C). In contrast, sarcomeric α-actinin was not detectable in these clusters by immunostaining. RT-PCR analysis confirmed that only the expression of neuronal markers was induced, whereas the expression of cardiac-specific genes was undetectable simultaneously (Fig. 3C, 4D). These results indicated that DAPT alone preferentially favored differentiation of iPSCs into the defined neuronal lineage.

**Figure 4:**
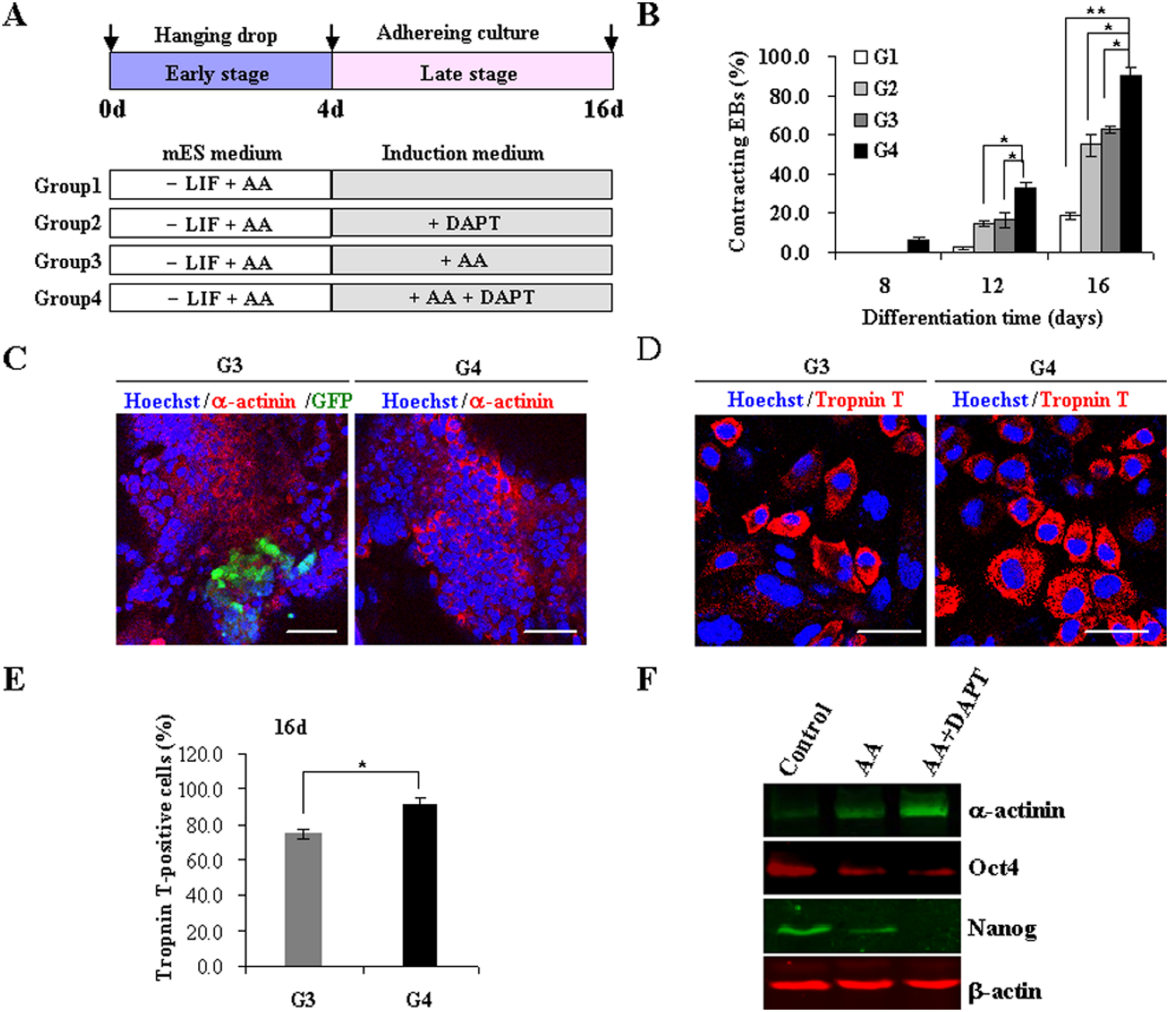
DAPT promotes cardiac differentiation from intermediate mesoderm. (**A**) Experimental strategies to optimize the induction procedure of four groups designated as G1 to G4. (**B**) The percentage of contracting EBs was counted at day 8, day 12 and day 16 among these groups. Data obtained from three independent experiments were shown as means ± s.d (*p<0.05, **p<0.01). (**C**) Immunofluorescence staining of α-actinin (Red) in AA (G3) and AA plus DAPT (G4) groups at day 16. GFP-positive expression indicated the existence of endogenous Oct4, representing undifferentiated iPSCs. Nuclei were counterstained with Hoechst33342 (Blue). Scale bar 50 µm. (D) Immunofluorescence staining of Tropnin T (Red) in AA (G3) and AA plus DAPT (G4) groups at day 16. Nuclei were counterstained with Hoechst33342 (Blue). Scale bar 50 µm. (**E**) Percentage of Troponin T-positive cells was calculated from at least five randomly selected fields at day 16. Data obtained from three independent experiments were shown as means ± s.d (*p<0.05). (**F**) The expression of α-actinin, Oct4, and Nanog was determined by Western blot analysis. Results are representative of three independent experiments.

### Sequential treatment of AA and DAPT promotes cardiac differentiation

For cardiomyogenesis, stem cells are compelled to undergo a consecutive array of differentiation, including mesoderm induction, specification of cardiac mesodermal cells towards cardiac progenitors, and the elaboration of functional beating cardiomyocytes. AA triggers the initiating step of mesoderm formation and promotes succeeding differentiation steps. Once cells enter the mesodermal stage, they may choose distinct paths to differentiate into cardiomyocytes, or the vascular smooth muscle and endothelial cells in response to environmental signals. Subsequently, we further investigated whether timely modulation of Notch signaling by DAPT affects cardiac differentiation from intermediate mesoderm.

After AA induction for 4 days at the early stage in suspension, EBs were allowed to attach for adherent growth and exposed to DAPT in the presence or absence of AA during the late-stage differentiation (Fig. 4A). DAPT daily supplemented from day 5 alone instructed the AA-pretreated iPSCs to differentiate into cardiomyocytes, as demonstrated by the enhanced amount of beating EBs compared with vehicle controls (Fig. 4B). It seems that AA plays an overwhelming role in decision between mesodermal and neuroectodermal cell fates. However, DAPT can facilitate the differentiation of mesodermal cells towards mature cardiomyocytes.

Intriguingly, the joint application of DAPT and AA during the late stage of differentiation greatly promoted the generation of beating cardiomyocytes (Fig. 4B, video S1 and S2). EBs induced by DAPT or AA alone generally started beating at day 12 of differentiation and continued to increase in number until day 16. In comparison, cotreatment of AA and DAPT resulted in an earlier emergence of beating clusters at day 8 and a marked increase in beating clusters, implying the faster development of cardiomyocytes. 54.7±5.6% and 62.8±2.2%, of the EBs in DAPT or AA groups respectively, developed contracting clusters up to 16 days examined. Whereas beating EBs in AA plus DAPT group were evidently elevated to 90.2±4.3% at the same time, reflecting a large-scale differentiation (Fig. 4B, *p*<0.05). Immunofluorescence staining against sarcomeric α-actinin and cardiac troponin T to specifically identify differentiated cardiomyocytes, further consolidated the synergic effect of AA combined with DAPT at day 16 compared with AA group (Fig. 4C and D). More than 91.6±3.6% cells expressed cardiac troponin T in AA plus DAPT group, compared to an average of 74.5±3.1% positive cells in AA group (Fig. 4E, *p*<0.05). Meanwhile, undifferentiated cells with endogenous Oct4 activity visualized by GFP expression, almost entirely vanished in AA plus DAPT group compared to AA group alone. Consistently, the cotreatment also increased the protein abundance of sarcomeric α-actinin indicating cardiomyocyte maturity (Fig. 4F). Collectively, DAPT administration beyond the mesoderm step has profound positive effects on the generation of contracting cardiomyocytes.

### DAPT promotes the generation of cardiac progenitors after mesoderm formation

**Figure 5:**
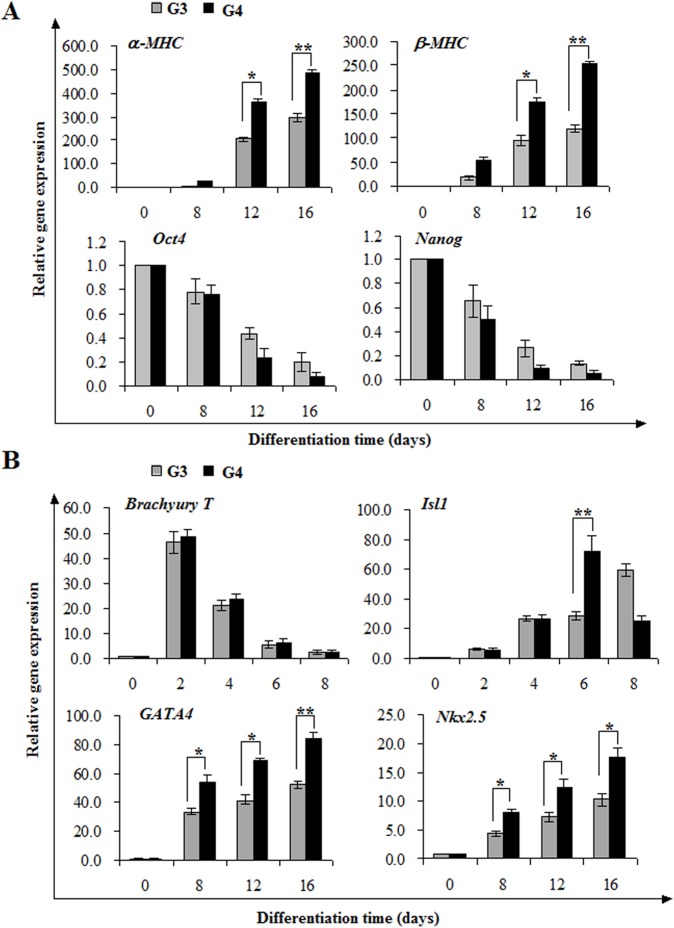
DAPT elevates the expression of cardiac transcriptional factors. (**A**) After AA induction for 4 days, iPSCs were subsequently treated by AA or AA plus DAPT. Real-time PCR analysis was performed to estimate the relative level of cardiac-specific and pluripotency-associated genes. (**B**) Real-time PCR analysis of mesoderm, cardiac progenitor and cardiac-specific transcription factor markers. Data are shown as relative gene expression with means ± s.d (n = 3) (*p<0.05, **p<0.01).

To illuminate the crucial stage of DAPT in promoting cardiac differentiation, we then performed real-time PCR to analyze the expression of typical genes marking distinct differentiation stages. The mRNA levels for cardiac structural and contractile proteins *α-MHC* and *β-MHC* increased significantly in EBs treated with AA followed by DAPT compared to control group treated with AA alone at day 12 to day 16. On the contrary, the expression of pluripotent markers *Oct4* and *Nanog* in cotreatment group declined more rapidly to a feeble level through successive differentiation (Fig. 5A). These results suggest that timely addition of DAPT gives rise to an accelerated differentiation of iPSCs into cardiomyocytes.

The cardiac differentiation program is tightly orchestrated by a hierarchical transcriptional network consisting of *Brachyury T*, *Isl1*, *GATA4* and *Nkx2.5*. *Brachyury T* is requisite for mesoderm formation and maturation. *Isl1* is among the earliest markers of the developing cardiac mesoderm, lying genetically upstream of the cardiac progenitor markers *GATA4* and *Nkx2.5* which control the onset of terminal differentiation. The synergy of *GATA4*, *Nkx2.5* and *Isl1* evoke the expression of cardiac structural and contractile proteins to generate beating cardiomyocytes. As shown in Fig. 5B, *Brachyury T* of both groups showed a nearly identical expression level during the examined periods. Compared to corresponding AA control group, *Isl1* in AA plus DAPT group was significantly increased from differentiation day 6 (2.6 fold) (Fig. 5B). As a result, there occurred a remarkable augment of *GATA4* and *Nkx2.5* mRNA over the following periods (*GATA4*: 1.6 fold, *p*<0.05; *Nkx2.5*: 1.8 fold, *p*<0.01 vs. AA alone at day 8).Therefore, our results indicate that DAPT can upregulate the expression of cardiac transcriptional factors to promote the generation of cardiac progenitors, thereby accelerating cardiac differentiation from intermediate mesoderm.

### Notch signaling is required for the early-stage cardiac differentiation

**Figure 6:**
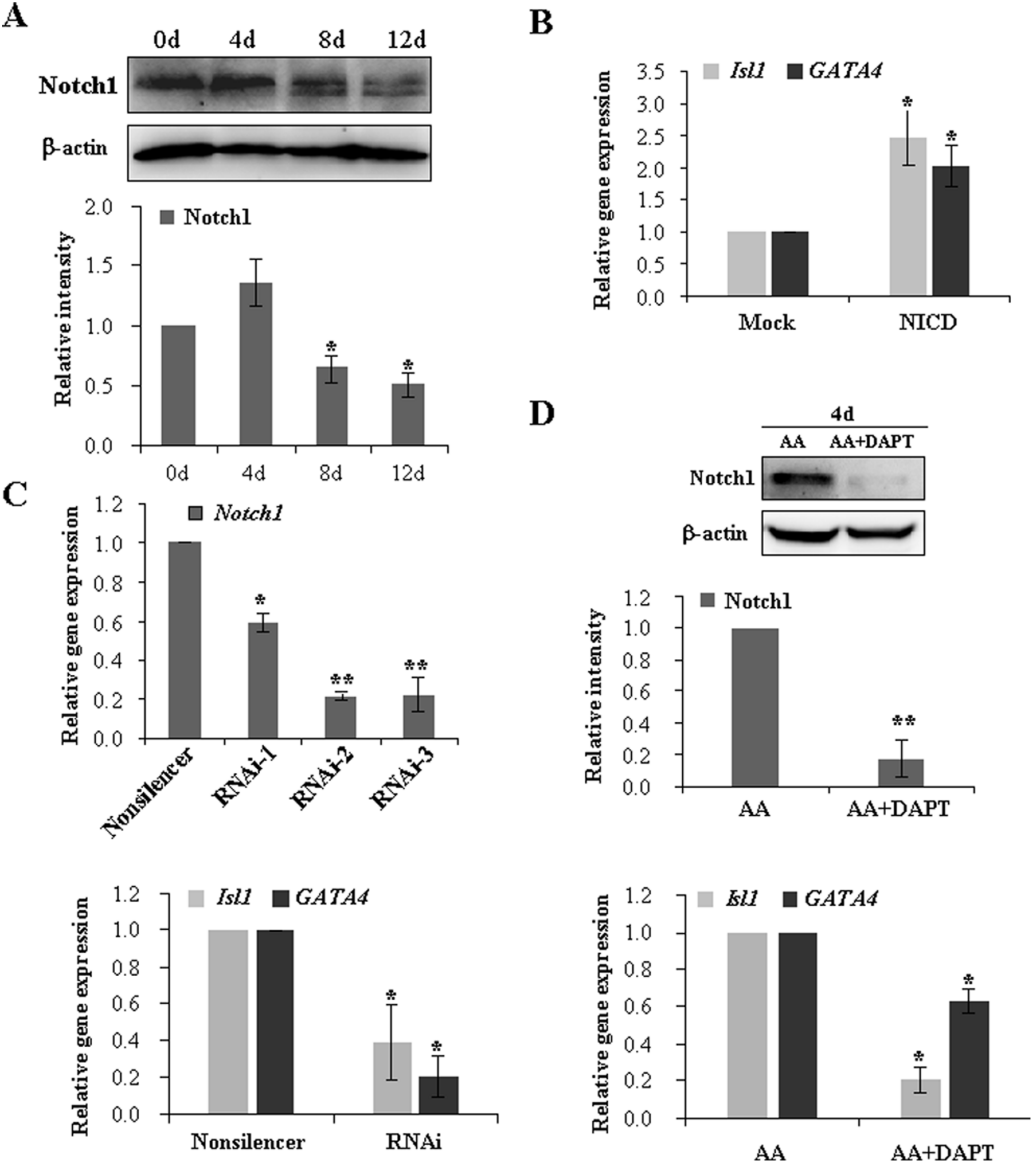
Notch1 promotes the expression of marker genes for the early-stage cardiac differentiation. (**A**) Immunoblot analysis showed the protein levels of Notch1 during iPSCs differentiation into cardiomyocytes. β-actin was used as an internal control (upper panel). The band intensity for Notch1 was normalized with that of β-actin and presented as relative intensity (lower panel) (*p<0.05 vs day 0). (**B**) Overexpression of NICD1 promoted the expression of *Isl1* and *GATA4*. After 24 h of transfection, the iPSCs were cultured in hanging drops with AA induction for 4 days. The mRNA level of *Isl1* and *GATA4* was measured and normalized to *18S* rRNA gene. (*p<0.05 vs mock group). (**C**) Real-time PCR was performed to determine RNAi efficiency mediated by three pairs of Notch1 RNAi duplexes (upper panel). The expression of *Isl1* and *GATA4* was evaluated under the condition of Notch1 knock-down by RNAi-2 duplexes (lower panel). The iPSCs were treated as the same method in B. (**D**) DAPT treatment during the early stage of cardiac differentiation inhibited the expression of *Isl1* and *GATA4.* The iPSCs were cultured with AA and DAPT in hanging drops for 4 days. Notch1 protein and the expression of *Isl1* and *GATA4* mRNA were measured by immunoblot (upper panel) or real-time PCR (lower panel), respectively. All data are shown as means ± s.d (n = 3) (*p<0.05, **p<0.01).

Given the observation that DAPT exerted different effects on the early- and late-stage cardiac differentiation, it was reasonable to hypothesize that Notch signaling may play a developmental stage-specific biphasic role in cardiomyogenesis. In fact, there were still substantial expression of Notch1 protein at day 4 of cardiac induction, which severely dropped during the late stage (Fig. 6A). It implicated that Notch signaling may be required for the early-stage cardiac differentiation, especially the process of mesoderm induction and maturation, whereas it may be deleterious for the late-stage differentiation after EB formation. Subsequently, we performed transient transfection with NICD1 plasmids and found that the overexpression of NICD1 indeed promoted the occurrence of *Isl1* and *GATA4* mRNA during the process of cardiac induction (Fig. 6B). We chemically synthesized three pairs of siRNA duplexes against Notch1 and identified the optimal siRNA sequence with about 80% silencing (RNAi-2). iPSCs were transfected with the optimal siRNA duplexes and allowed to form EBs for cardiac differentiation in the presence of AA. In contrast, blockade of Notch signaling by specific siRNA against Notch1 decreased the expression of *Isl1* and *GATA4* (Fig. 6C). Meanwhile, we also observed that DAPT administration during the formation of EBs depressed the expression of Notch1 protein, thereby attenuating the efficiency of early differentiation (Fig. 6D). Thus, we concluded that Notch signaling is essential for the early-stage cardiac differentiation.

### DAPT modulates Notch/Wnt/BMP signalings to promote the late-stage cardiac differentiation

**Figure 7:**
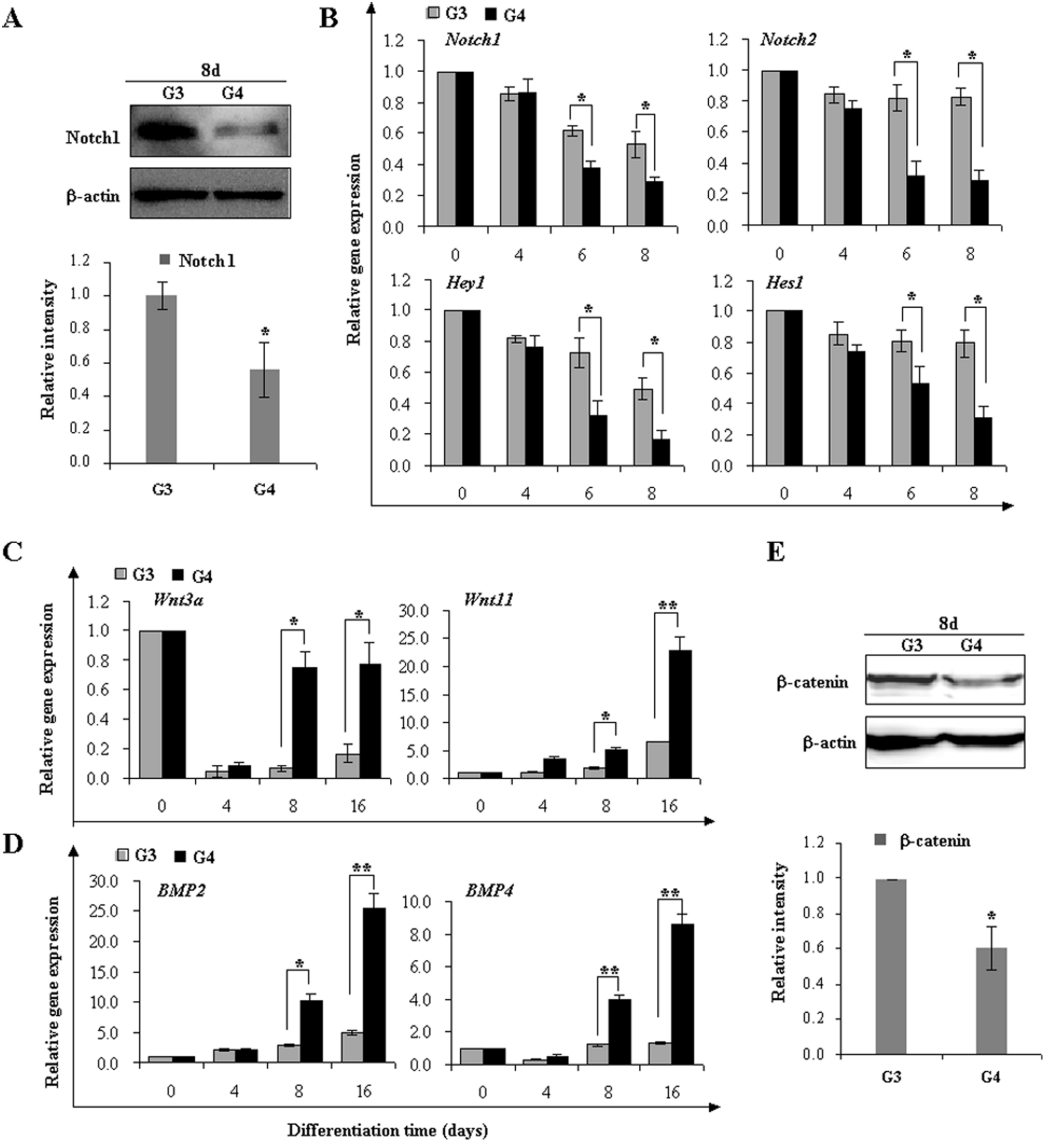
DAPT promotes the late-stage cardiac differentiation by Notch inhibition. (**A**) Immunoblot analysis demonstrated the protein amount of Notch1. After AA induction for 4 days, iPSCs were subsequently treated by AA in the presence or absence of DAPT during the cardiomyocyte differentiation. Cell lysates were analyzed for Notch1 or β-actin (upper panel). Normalized densitometric quantification of Notch1 bands was performed with images of three independent experiments (lower panel). (**B**) Real-time PCR analysis was performed to determine the expression of Notch family genes expression at indicated time points. (**C**) and (**D**) Real-time PCR analysis of *Wnt3a*, *Wnt11, BMP2, and BMP4* expression. (**E**) Immunoblot analysis demonstrated the protein amount of β-catenin. β-actin was used as the internal control. All data are shown as means ± s.d (n = 3) (*p<0.05, **p<0.01).

Subsequently, we sought to determine whether DAPT favors the late-stage cardiac differentiation by inhibiting Notch signaling. As mentioned above, DAPT alone or combined with AA facilitated the late-stage differentiation. In parallel, DAPT treatment significantly decreased the protein amount of Notch1 (Fig. 7A). Real-time PCR assay showed a simultaneous and persistent decrease of *Notch1*, *Notch2*, *Hes1*, and *Hey1* in expression after EBs exposure to DAPT at day 6 and day 8 in AA plus DAPT group compared with AA group (Fig. 7B). As *Hes1* and *Hey1* were target genes of canonical Notch/RBP-J pathway, their downregulation reflected the inhibition of endogenous Notch activity, probably resulting from decreased expression of *Notch1* and *Notch2*. Therefore, pharmaceutical modulation provided strong evidences implicating that Notch signaling deteriorates the late-stage cardiac differentiation. Taken together, it is rational that temporal-dependent inhibition of Notch signaling by DAPT should yield different outcomes of cardiac differentiation. Our findings shed light on possible pathways to generate cardiomyocytes via stage-specific Notch modulation.

With respect to cardiac differentiation, there exists a reciprocal crosstalk between Notch and other developmental signals. Subsequently, we assessed the effect of DAPT on Wnt and BMP signaling pathways. As shown in Fig. 7C, DAPT significantly increased the expression of canonical *Wnt3a* and noncanonical *Wnt11*. DAPT also elicited over a 3.6- and a 5.2-fold increase in *BMP2* expression as well as a 3.3- and a 6.5-fold in *BMP4* expression relative to controls at day 8 and day 16 (Fig. 7D, *p*<0.01). It is worth noting that canonical Wnt signaling has a biphasic effect on cardiomyogenesis, and that its blockade in stem cells seems to be a prerequisite for terminal differentiation into mature cardiomyocytes at the late stage, while noncanonical Wnt11 and BMP2/4 readily facilitate cardiac differentiation in a wide variety of *in vitro* model systems. Despite the upregulation of *Wnt3a*, there occurred a significant decrease in β-catenin expression after DAPT treatment, reflecting the ultimate inhibition of canonical Wnt pathway (Fig. 7E). Thus, DAPT impacts cardiomyogenesis via an integrated modulation of Notch and other signaling pathways.

## Discussion

In this report, we provided a simple and user-friendly protocol for practical production of cardiomyocytes derived from iPSCs by a two-step induction with small molecules. A sequential combination of AA and DAPT efficiently promotes cardiac differentiation of iPSCs.

Cardiomyogenesis is a tightly orchestrated process integrating numerous signaling pathways and transcription factors in a synergistic manner. A crowd of signaling pathways, such as BMP, Wnt, Notch, FGF and Hedgehog, has been implicated in cardiac specification and terminal differentiation. Small molecules, pharmacologically modulating specific signaling pathways or other mechanisms, are an alternative approach for manipulating stem cells for cardiac regeneration, substituting for growth factors or signaling molecules. Targeting Notch signaling by small molecules apparently is a promising approach for cardiac induction.

During heart development, Notch pathway plays versatile roles in several processes, such as progenitor proliferation, terminal differentiation, tissue patterning and boundary formation. Ample models of Notch pathway activation or inhibition from gene-targeted mice have revealed essential roles for Notch during ventricular, atrioventricular canal, and outflow tract development. At the same time, both loss- and gain-of function of the Notch pathway results in defects in heart development [14], [16], [25], [26].

Genetic and pharmacological experiments within pluripotent stem cells disclosed contradictory roles of Notch pathway in cardiac differentiation. Several lines of evidence pointed to an inhibitory role of Notch signaling in cardiac differentiation. For instance, Notch1 activation in *Xenopus* embryo decreased myocardial gene expression in the early heart field. In the developing heart of chick embryo, constitutive activation of Notch signaling also inhibited cardiac differentiation [27]. Although specific deletion of *RBP-J* in mice resulted in embryonic lethality likely due to pericardial edema, *RBP-J* deficient ESCs favored the specification towards mesodermal lineages and generated more cardiomyocytes than wild-type counterparts [28]. ESCs deficient in *Notch1* exhibited an elevated potential of cardiac differentiation [17]. Paradoxically, there is also opposite evidence that Notch activation can promote cardiac specification in multiple stem cells, including circulating endothelial progenitor cells, mesenchymal stem cells, and hemangioblasts [19]–[21]. Although this is highly controversial about the outcomes of Notch activation, emerging evidence unequivocally supports that small molecules targeting Notch signaling effectively favors cardiomyogenesis. For instance, persistent addition of a γ-secretase inhibitor (GSI) to block Notch signaling alone failed to affect ESC differentiation, but accelerated cardiac mesoderm differentiation when simultaneously plus reduced-volume culture medium [18].

Herein, our results help to resolve some of the discrepancies between these studies, indicating that Notch pathway play a stage-specific biphasic role in cardiomyogenesis. As demonstrated by NICD1 overexpression, the activation of Notch signaling at the early stage promoted cardiac differentiation. In contrast, Notch1 inhibition by specific siRNA did the opposite. Pharmacological blockade of Notch activation by DAPT further strengthened this conclusion. DAPT administration after EB formation promoted cardiac specification via facilitating the formation and maturity of cardiac mesoderm, demonstrated by enhanced the expression of cardiac marker genes such as *Isl1*, *GATA4*, and *Nkx2.5*. It seemed that Notch activation favors the mesoderm induction at the early stage, but deteriorates the formation and maturation of cardiac progenitors at the late stage of cardiac differentiation. Just like Wnt pathway, there is an initial activation of the canonical Wnt pathway, followed by repression during cardiomyogenesis [29]. Meanwhile, there was also a relevant evidence exhibiting the biphasic role of Notch pathway during cardiac differentiation of P19CL6 cells [30]. Therefore, these seemingly contradictory results may derive from the heterogeneity of stem cells from different sources, with Notch playing disparate roles within different cellular models and/or different stages of differentiation.

Based on these novel findings, it will be practicable to generate large quantities of cardiomyocytes via stage-specific modulation of Notch signaling. DAPT, a small molecule inhibitor of γ-secretase, efficiently prevents activation of Notch signaling. Previous studies showed that ESCs were preferentially apt to neural commitment upon Notch inhibition by DAPT [31], [32]. In this study, DAPT alone facilitated the neural differentiation of iPSCs forming EBs in a hanging drop system, characterized by the positive expression of Nestin and Pax6. Nevertheless, the timed treatment of AA and DAPT after EBs formation induced substantial differentiation of iPSCs towards myocardial lineage. Apparently, DAPT alone is insufficient to initiate cardiomyogenesis, coincident with that Notch1 RNAi antagonized the cardiac differentiation at the early stage. But it can bring about an additive or reinforcing effect to accelerate the further differentiation and maturation of committed precursors, due to its inhibition on Notch signaling. Under our culture conditions, the synergy of AA and DAPT produced highly efficient cardiomyocyte differentiation from iPSCs.

Beyond the inhibition of canonical Notch signaling, γ-secretase inhibitors may exert more extensive effect on cardiomyogenesis. Firstly, there exist mutual cross-talks of Notch with other transduction pathways, resulting in a synergy or antagonism [15]. Herein, we also found that DAPT treatment increased the expression of *BMP2/4* and *Wnt11*, which were verified to be promoting signals for cardiac differentiation. On the opposite of our prediction, DAPT enhanced the expression of *Wnt3a*, which activates canonical Wnt pathway and is believed to be adverse for the late-stage differentiation of cardiomyocytes. The upregulation of canonical *Wnt3a* might be a side-effect of DAPT, which converged with other signaling pathways to modulate cardiac differentiation and did not alter the integrated outcome of DAPT treatment. Secondly, recent studies suggest that Notch can non-canonically exert its biological functions by antagonizing Wnt/β-catenin signaling independent of Notch ligand-dependent cleavage [33], [34]. However, γ-secretase inhibitors can also lower the protein level of β-catenin in various cell lineages. It seems that γ-secretase inhibitors can mimic the activation of non-canonical Notch signaling. In this study, we also observed that DAPT decreased the expression of β-catenin, which represents the inhibition of canonical Wnt pathway. Despite its positive effect on *Wnt3a* expression, the overall effect of DAPT ultimately led to the inhibition of canonical Wnt pathway. Therefore, DAPT treatment should exert a comprehensive effect on multiple signaling pathways involved in cardiac differentiation.

In this study, we developed a two-step induction procedure optimized for cardiac differentiation of iPSCs via Notch inhibition. Most of the existing cardiac induction protocols require an elaborate switch of culture medium, and supplement of inducers at the accurate timing and concentration. The consequence of targeting signaling pathways displays an affluent versatility depending on the timing, dosage, and duration of treatment as well as cellular context. BMP pathway is believed to promote cardiac specification, whereas Wnt signaling has biphasic effects, functioning as either an agonist or antagonist depending on the distinct stage of differentiation [29]. However, a discontinued inhibition of BMP signaling by dorsomorphin in a temporal-dependent manner also drove robust cardiac induction [35]. A timely administration of a Wnt activator before the formation of mesoderm progenitor cells led to a marked increase in production of cardiomyocytes [36]. Additionally, SB203580, a specific p38 MAP kinase inhibitor, enhanced the differentiation of ESCs into cardiomyocytes by favoring early mesoderm formation at low concentrations, but strongly attenuated cardiomyogenesis at high concentrations [37]. Consistent with previous reports, our results also showed that differentiation stage-based manipulation is required for targeting signaling pathways to generate cardiomyocytes.

Overall, our results indicate that Notch pathway exhibits biphasic and antagonistic effects on cardiomyogenesis. Timely modulation of Notch pathway by DAPT or other small molecules could allow efficient, inexpensive and reproducible generation of cardiomyocytes from iPSCs or other tissue-derived cells in a simple and rapid manner.

## Supporting Information

Figure S1 **Morphology and Characterization of iPSCs.** **(A)** Cell morphology changes in the iPSC derivation process at day 0, day 5 and day 12 after Dox induction. The colonies were stained positive for alkaline phosphatase. Scale bar 100 µm. **(B)** Two fully reprogrammed iPS colonies stably expressing GFP after withdraw of Dox were picked, designated as iPS-C2 and iPS-C4. Top, phase contrast view; bottom, Oct4-GFP colonies (Green).
(TIF)

Table S1 **Primers used for RT-PCR.**
(DOC)

Table S2 **Primers used for RT-PCR and real-time PCR.**
(DOC)

Table S3 **Primers used for real-time PCR.**
(DOC)

Video S1 **Video record of beating cardiomyocytes from iPSC cells induced with AA (G3 group).**
(AVI)

Video S2 **Video record of beating cardiomyocytes from iPSC cells induced with AA plus DAPT (G4 group).**
(AVI)
