# Supplementary figures and images for: Timely Inhibition of Notch Signaling by DAPT Promotes Cardiac Differentiation of Murine Pluripotent Stem Cells
Source: PLoS One. 2014 Oct 14;9(10):e109588. doi: 10.1371/journal.pone.0109588 (PMC4196912; doi:10.1371/journal.pone.0109588)

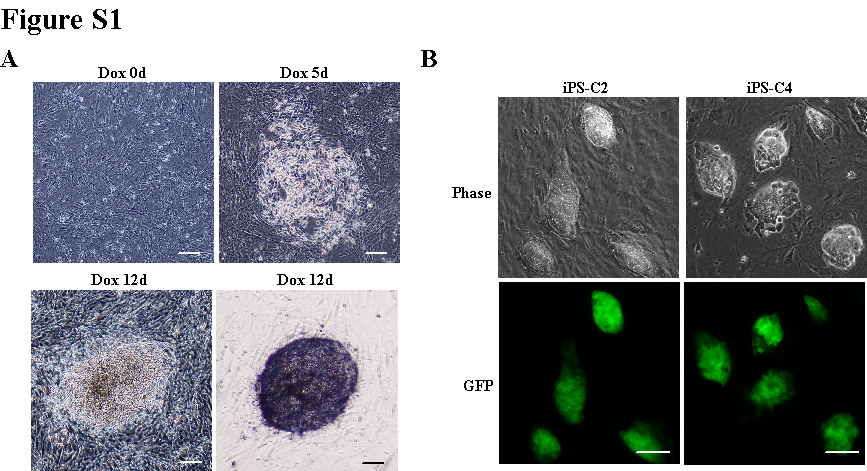

Supplement: Figure S1 — Morphology and Characterization of iPSCs. (A) Cell morphology changes in the iPSC derivation process at day 0, day 5 and day 12 after Dox induction. The colonies were stained positive for alkaline phosphatase. Scale bar 100 µm. (B) Two fully reprogrammed iPS colonies stably expressing GFP after withdraw of Dox were picked, designated as iPS-C2 and iPS-C4. Top, phase contrast view; bottom, Oct4-GFP colonies (Green). (TIF) [file pone.0109588.s001.tif]
