# Supplementary material for: Timely Inhibition of Notch Signaling by DAPT Promotes Cardiac Differentiation of Murine Pluripotent Stem Cells
Source: PLoS One. 2014 Oct 14;9(10):e109588. doi: 10.1371/journal.pone.0109588 (PMC4196912; doi:10.1371/journal.pone.0109588)
Supplement: Table S1 — Primers used for RT-PCR. (DOC) [file pone.0109588.s002.doc]

**Supplemantal information**

**Table S1 Primers used for RT-PCR**

| **Gene** | **Primer Sequence(5’ to 3’)** | **AT(°C)** | **Product(bp)** |
| --- | --- | --- | --- |
| *Pou5f1* | F-AGTTGGCGTGGAGACTTTGC  R-CAGGGCTTTCATGTCCTGG | 58 | 160 |
| *Sox2* | F-GCGGAGTGGAAACTTTTGTCC  R-CGGGAAGCGTGTACTTATCCTT | 58 | 157 |
| *Nanog* | F-CCTGATTCTTCTACCAGTCCCA  R-GGCCTGAGAGAACACAGTCC | 58 | 123 |
| *Exo-Pou5f1* | F-GGCTGTATCCTTTCCTCTGC  R-GCATTAAAGCAGCGTATCCA | 56 | 472 |
| *Exo-Sox2* | F-ACCTCTTCCTCCCACTCCAG  R-GTTGCGTCAGCAAACACAGT | 56 | 460 |
| *Exo-Klf4* | F-ACACCTGCGAACTCACACAG  R-GGCATTAAAGCAGCGTATCC | 56 | 435 |
| *Exo-cMyc* | F-TGTCCATTCAAGCAGACGAG  R-GTTGCGTCAGCAAACACAGT | 56 | 580 |
| *Cripto* | F-ATGGACGCAACTGTGAACATGATGTTCGCA  R- CTTTGAGGTCCTGGTCCATCACGTGACCAT | 58 | 174 |
| *Dppa5a* | F- ATGATGGTGACCCTCGTGAC  R- ACCTCGATAAGTTCTTCGGGAG | 58 | 174 |
| *Eras* | F- TGCCTACAAAGTCTAGCATCTTG  R- CTTTTACCAACACCACTTGCAC | 58 | 157 |
| *FGF4* | F- GGGCATCGGATTCCACCTG  R- GCTGCTCATAGCCACGAAGAA | 58 | 154 |
| *UTF1* | F- TGTCCCGGTGACTACGTCT  R- CCCAGAAGTAGCTCCGTCTCT | 58 | 135 |
| *Rex1* | F-ACGAGTGGCAGTTTCTTCTTGGGA  R- TATGACTCACTTCCAGGGGGCACT | 58 | 209 |
| *GAPDH* | F-CATGTTCCAGTATGACTCCACTC  R-GGCCTCACCCCATTTGATGT | 58 | 136 |
