# Supplementary material for: Timely Inhibition of Notch Signaling by DAPT Promotes Cardiac Differentiation of Murine Pluripotent Stem Cells
Source: PLoS One. 2014 Oct 14;9(10):e109588. doi: 10.1371/journal.pone.0109588 (PMC4196912; doi:10.1371/journal.pone.0109588)
Supplement: Table S2 — Primers used for RT-PCR and real-time PCR. (DOC) [file pone.0109588.s003.doc]

**Supplemantal information**

**Table S2 Primers used for RT-PCR and real-time PCR**

| **Gene** | **Primer Sequence(5’ to 3’)** | **AT(°C)** | **Product(bp)** |
| --- | --- | --- | --- |
| *GATA4* | F-CACCCCAATCTCGATATGTTTGA  R-GGTTGATGCCGTTCATCTTGT | 58 | 151 |
| *Nkx2.5* | F-CAAGTGCTCTCCTGCTTTCC  R- GGCTTTGTCCAGCTCCACT | 56 | 136 |
| *Mef2c* | F- GTCAGTTGGGAGCTTGCACTA  R- CGGTCTCTAGGAGGAGAAACA | 58 | 112 |
| *Isl1* | F- CAGTCCCAGAGTCATCCGAGT  R- TGGGTTAGCAGTTTTGTCGTT | 58 | 115 |
| *α-MHC* | F- GCCCAGTACCTCCGAAAGTC  R- GCCTTAACATACTCCTCCTTGTC | 58 | 110 |
| *β-MHC* | F- ACAACCCCTACGATTATGCGT  R- ACGTCAAAGGCACTATCCGTG | 58 | 100 |
| *Brachury T* | F-GCTTCAAGGAGCTAACTAACGAG  R-CCAGCAAGAAAGAGTACATGGC | 57 | 117 |
| *Nothch 1* | F-CCCTTGCTCTGCCTAACGC  R-GGAGTCCTGGCATCGTTGG | 57 | 162 |
| *Nothc 2* | F- ATGTGGACGAGTGTCTGTTGC  R- GGAAGCATAGGCACAGTCATC | 57 | 146 |
| *Hey1* | F-CAGCCCTTCGCAGATGCAA  R-CCAATCGTCGCAATTCAGAAAG | 57 | 101 |
| *Hes1* | F-ATAGCTCCCGGCATTCCAAG  R-GCGCGGTATTTCCCCAACA | 57 | 133 |
| *Pax6* | F- TACCAGTGTCTACCAGCCAAT  R- TGCACGAGTATGAGGAGGTCT | 57 | 194 |
| *Nestin* | F- CCCTGAAGTCGAGGAGCTG  R- CTGCTGCACCTCTAAGCGA | 57 | 166 |
| *18S rRNA* | F- GTAACCCGTTGAACCCCATT  R-CCATCCAATCGGTAGTAGCG | 58 | 151 |
