# Supplementary material for: Timely Inhibition of Notch Signaling by DAPT Promotes Cardiac Differentiation of Murine Pluripotent Stem Cells
Source: PLoS One. 2014 Oct 14;9(10):e109588. doi: 10.1371/journal.pone.0109588 (PMC4196912; doi:10.1371/journal.pone.0109588)
Supplement: Table S3 — Primers used for real-time PCR. (DOC) [file pone.0109588.s004.doc]

**Supplemantal information**

**Table S3 Primers used for real-time PCR**

| **Gene** | **Primer Sequence(5’ to 3’)** | **AT(°C)** | **Product(bp)** |
| --- | --- | --- | --- |
| *BMP2* | F-TCTTCCGGGAACAGATACAGG  R-TGGTGTCCAATAGTCTGGTCA | 58 | 126 |
| *BMP4* | F-TTCCTGGTAACCGAATGCTGA  R-CCTGAATCTCGGCGACTTTTT | 58 | 114 |
| *Wnt3a* | F-AATTTGGAGGAATGGTCTCTCGG  R-CAGCAGGTCTTCACTTCACAG | 58 | 175 |
| *Wnt11* | F-ATGCGTCTACACAACAGTGAAG  R-GTAGCGGGTCTTGAGGTCAG | 58 | 159 |
